# Spontaneous Salivation—A Suggestion

**Published:** 1863-10

**Authors:** 


### SPONTANEOUS SALIVATION—A SUGGESTION.

Editor Buffalo Medical and Surgical Journal:

Sir:—A good deal having been said in the Medical Journals, as well as in the newspapers, regarding the abuse of mercury by the medical officers of the army, and especially since the late order of the Surgeon-General on the subject, it has struck me that perhaps many cases have been reported of mercurial ptyalism which were in reality cases of spontaneous salivation. This is mentioned as an idiopathic affection by medical authors, but I believe is somewhat rare. While serving with the Twenty-first Regiment in August, 1862, I met several cases, and as they served to prove to me at least, that the disease was not a myth, and that it might exist in the army, I am inclined to the belief that it may have often been mistaken for the mercurial affection. I regret that I cannot give a detailed history of the cases, but perhaps such a brief account of them as I can give from memory may not be uninteresting to those who are discussing the question.

In August, 1862, while we were encamped on the since celebrated "Maryland Heights," near Fredericksburg, I discovered symptoms of severe salivation in a patient in the regimental hospital; as I had been giving him some calomel I of course attributed it to that, and such was the view taken of it by Surgeon Wilcox, (then Chief Surgeon of the Brigade,) at his visit of inspection, where he saw the case. My only difficulty was to account for so severe symptoms from such a small quantity of the drug as he had taken. A few days later I found the same affection in a man who had taken no mercurial at all. I cannot now recall the precise number of cases which occurred, but think there were about half a dozen, some of whom had and some had not taken calomel, or *pil hydrargyri*, but none of whom had been subjected to a "mercurial course," or had taken more than a very small amount of the remedy. As all the patients were well known to me from long association, I felt sure that, unless in the first case, there could be no such idiosyncracy as sometimes exists and renders even a small amount of mercury a poison, so I put mercury out of the question, even in the cases of those who had taken it; in the cases of those who had not, there could of course be no doubt. Nor was there, I think, any mistake in the diagnosis; it seemed very clear to both Surgeon Wilcox and myself.

Of the causes of this disease I can say but little, and have no theory to offer. Fevers of various grades, and affections of the alimentary canal, were prevalent, from the effects of the season and other causes. I believe no case occurred of a man otherwise healthy, being affected with ptyalism, nor could we trace it to any drug, though many of the list are accused of causing it. Scurvy was also quite out of the question, as the men were well nourished, and there were no signs of it. As to treatment, we found a gargle of brandy and water most effectual, though other plans were tried.

If it were generally known that salivation is not always a sign of overdosing by mercury, much of the obloquy heaped upon medical officers might perhaps be averted.

Very truly yours, JOSEPH A. PETERS.

---

CRESTLINE, CRAWFORD CO., Ohio, Sept. 11, 1863.

Editor Medical and Surgical Journal:

Dear Sir:—Will you please send my number of the *Buffalo Medical and Surgical Journal* to me at Crestline, Crawford Co., Ohio? Also, for the good of the profession at large, please publish in the Journal the following recipe, which is now being sold by a celebrated traveling Doctor.

For the common good of the profession and humanity in general, I have purchased it, and wish to make it public. The following is a true copy of the original. E. BOOTH.

"Medicine for to distract the Reumatism, Pains and toothake, Backake and boils, strains for man & horses & many more sores: Take one quart of good Old Rye Whiskey & add in the bottle One oz of campfire, six pots of Red pepper, six cents worth of gloves, 5 cents worth of cinnamund, 3 cents worth of shaven sope, One table spoonful of salt. Add them all in the bottle together & set the bottle in the sun 9 days before useing & where the pain is grease it well Evening and Morning. Dont give up soon. Shak the bottle 2 or 3 times a day.

50 cents for a reseat. It is a sure cure."

